# Genes associated with cognitive performance in the Morris water maze: an RNA-seq study

**DOI:** 10.1038/s41598-020-78997-6

**Published:** 2020-12-16

**Authors:** Vasiliy V. Reshetnikov, Polina E. Kisaretova, Nikita I. Ershov, Anastasia S. Shulyupova, Dmitry Yu. Oshchepkov, Natalia V. Klimova, Anna V. Ivanchihina, Tatiana I. Merkulova, Natalia P. Bondar

**Affiliations:** 1 Institute of Cytology and Genetics, Siberian Branch of Russian Academy of Sciences (SB RAS), Novosibirsk, Russia; 2 Novosibirsk State University, Novosibirsk, Russia

**Keywords:** Learning and memory, Molecular neuroscience, Transcriptomics

## Abstract

Learning and memory are among higher-order cognitive functions that are based on numerous molecular processes including changes in the expression of genes. To identify genes associated with learning and memory formation, here, we used the RNA-seq (high-throughput mRNA sequencing) technology to compare hippocampal transcriptomes between mice with high and low Morris water maze (MWM) cognitive performance. We identified 88 differentially expressed genes (DEGs) and 24 differentially alternatively spliced transcripts between the high- and low-MWM-performance mice. Although the sets of DEGs and differentially alternatively spliced transcripts did not overlap, both were found to be enriched with genes related to the same type of biological processes: trans-synaptic signaling, cognition, and glutamatergic transmission. These findings were supported by the results of weighted-gene co-expression network analysis (WGCNA) revealing the enrichment of MWM-cognitive-performance-correlating gene modules with very similar Gene Ontology terms. High-MWM-performance mice manifested mostly higher expression of the genes associated with glutamatergic transmission and long-term potentiation implementation, which are processes necessary for memory acquisition and consolidation. In this set, there were genes participating in the regulation of trans-synaptic signaling, primarily AMPA receptor signaling (*Nrn1*, *Nptx1*, *Homer3*, *Prkce*, *Napa*, *Camk2b*, *Syt7*, and *Nrgn*) and calcium turnover (*Hpca*, *Caln1*, *Orai2*, *Cpne4*, and *Cpne9*). In high-MWM-performance mice, we also demonstrated significant upregulation of the “flip” splice variant of *Gria1* and *Gria2* transcripts encoding subunits of AMPA receptor. Altogether, our data helped to identify specific genes in the hippocampus that are associated with learning and long-term memory. We hypothesized that the differences in MWM cognitive performance between the mouse groups are linked with increased long-term potentiation, which is mainly mediated by increased glutamatergic transmission, primarily AMPA receptor signaling.

## Introduction

Learning and memory are among the most crucial processes in the brain. Without them, living creatures would have a set of only simple reflexes, behave stereotypically, and would be unable to use lived experience for adaptation to new conditions^1–3^. It should be noted that memory is an essential factor for learning because it helps to gather and retrieve information after the learning process^4,5^. Research into the mechanisms of learning and memory not only is one of the central tasks of neuroscience but also is important for the development of new (and improvement of existing) therapeutic approaches to cognitive disorders as well as mood disorders because many psychiatric disorders involve disturbances of cognitive processes^6–9^.

The hippocampus is a brain region critical for learning and memory processes, including spatial memory and flexible memory of past events^10–13^. The integrity of the hippocampus is necessary for learning and memory (including social memory), social behavior, and anxiety^14–16^. Additionally, reduced hippocampal function is strongly related to cognitive dysfunction in some disorders such as autism^7^, schizophrenia^9^, and major depressive disorder^6,14,17^.

Transcription is the first and most important stage of the molecular events underlying the transfer of information encoded in the genome to the phenotypic level. Accordingly, to date, there have been many studies on the transcriptional changes occurring during various processes in the nervous system in health and in various neurological diseases. Originally, both this research and studies on the expression of genes in the course of other physiological and pathological processes were focused on the investigation of individual genes. The advent of genome-wide techniques (first, oligonucleotide or cDNA microarrays, and then, next-generation sequencing approaches) has taken this research to a new level by enabling investigators to study complex gene networks simultaneously instead of one or several genes at a time^18^.

**Figure 1:**
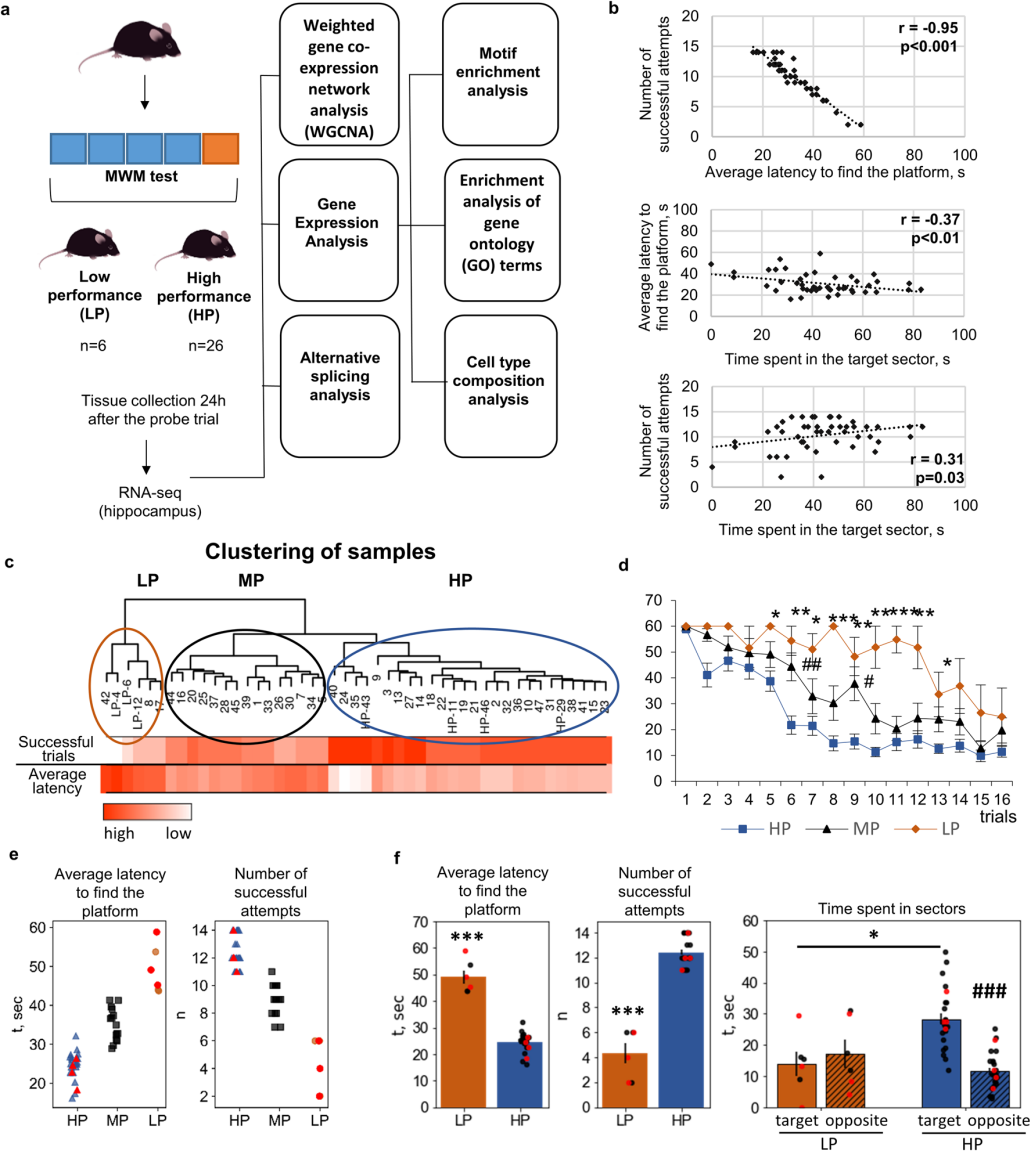
MWM cognitive performance. (**a**) Experimental design. (**b**) The correlation among the parameters of the MWM test among all the mice. (**c**) Hierarchical clustering of samples based on the number of successful attempts to find the platform and the average latency to find the platform in the MWM test. Mice were subdivided into three groups: low (LP), medium (MP), and high (HP) MWM performance. Hippocampus samples from the mice labeled as LP or HP were used in the subsequent transcriptome analysis. (**d**) The learning curve in the MWM test shows the change in latency to find the platform during 16 training trials (four trials a day for 4 days) in groups LP, MP, and HP. (**e**) Scatter plots showing the distribution of numbers of successful attempts to find the hidden platform and the distribution of latency times to find the platform among the animals. (**f**) A comparison of parameters of the MWM test between groups LP and HP. Data are presented as mean ± SEM (**d**,**f**), *p < 0.05, **p < 0.01, ***p < 0.001 as compared with the LP group, ^###^p < 0.001 as compared with the Target sector.

In our study, we applied a next-generation-sequencing–based technology (high-throughput mRNA sequencing; RNA-seq) to identify in adult male mice specific genes and neuronal networks in the hippocampus that are responsible for cognitive performance on the Morris Water Maze (MWM) test. This test allows to assess the capacity for spatial learning and long-term memory. We compared transcriptomes of two contrasting groups of animals: showing either high MWM performance (better cognitive metrics and shorter latency to find the platform) or low MWM performance (worse cognitive metrics and longer latency to find the platform). For analysis of the RNA-seq data, we performed (1) a classic gene expression analysis to identify differentially expression genes (DEGs) in combination with detection of differential alternative splicing events and (2) the search for the clusters of co-expressed genes by weighted-gene co-expression network analysis (WGCNA). In addition, via various bioinformatic approaches, we tried to find a master regulator of the genes underlying more successful learning in a spatial memory test (the design of experiment is shown on Fig. 1a).

## Results

### Variation of learning and long-term memory performance among C57BL/6 mice

The use of a large group of animals (47 mice) in the MWM test allowed to uncover individual features of cognitive performance. To assess MWM cognitive performance, we used two metrics of learning (the number of successful attempts and average latency to find the platform during 16 attempts) and one metric of long-term memory (time spent in the target sector in a probe trial). As expected, the metrics of learning highly correlated with each other (r =  − 0.95,* p* < 0.001, Fig. 1b). Correlations were found between the learning variables and long-term memory performance too: time spent in the target sector significantly correlated with the number of successful attempts (r = 0.31,* p* < 0.01) and average latency to find the platform (r =  − 0.37, *p* < 0.01).

We applied the cluster analysis for the classification of the mice into groups based on their learning abilities (i.e., variables “average latency to find the platform” and “the number of successful attempts to find the platform”). The animals were subdivided into three groups: low (LP; six animals), moderate (MP; 15 animals), and high (HP; 26 animals) cognitive performance on the MWM test (Fig. 1c). All mice of the LP group made no more than six successful attempts to find the platform in training trials, and the average latency to find the platform was > 40 s. All mice of the HP group made ≥ 11 successful attempts to find the platform, and the average latency to find the platform was < 32 s (Fig. 1e). Figure 1d presents differences in the latency to find the platform among the three groups. The Kruskal–Wallis test revealed significant differences among the groups from trial 5 to trial 13 (Fig. 1d). The pairwise Mann–Whitney *U* test indicated that HP mice were quicker at finding the platform and made a greater number of successful attempts as compared to LP mice (p < 0.001; Fig. 1f). In the probe trial, only HP mice showed a preference for the Target sector over the Opposite sector (p < 0.001; Fig. 1f), and HP mice had better memory of the location of the platform than LP mice did (p < 0.01; Fig. 1f). These results indicate that mice of the HP group learned faster to find the hidden platform: already after the 8^th^ trial, their latency period stopped changing, evidently reaching its minimum. Mice of the LP group attained such values of average latency only by the last trial, indicating slower learning. Nonetheless, in the probe trial, only HP mice preferred the Target sector, suggesting that long-term memory was forming.

### Corticosterone levels

**Figure 2:**
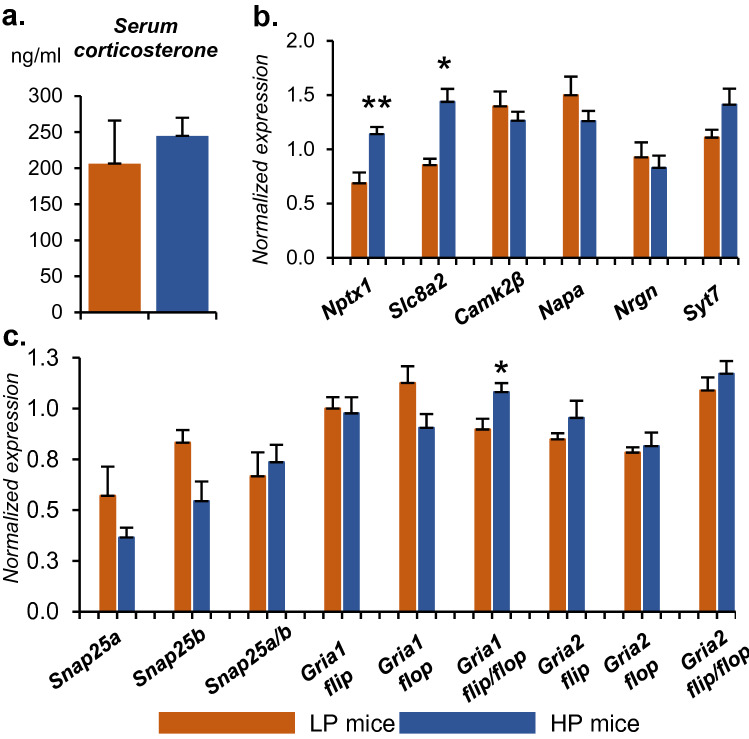
qPCR analysis for validation of DEGs and DASGs. (**a**) Levels of serum corticosterone. (**b**) Relative expression of genes related to synaptic plasticity. (**c**) Relative expression of alternatively spliced transcripts. Data are presented as mean ± SEM (**a**–**c**), *p < 0.05, **p < 0.01 as compared to the LP group (Student’s *t* test).

Because the MWM test by itself is a strong stressor^19^, we evaluated its influence on the serum corticosterone level mice of groups HP and LP at 1 day after the end of testing, at the time point of tissue collection. Corticosterone levels were not significantly different among the groups (p = 0.491; Fig. 2a).

### Learning abilities are reflected in hippocampal gene expression

The most different groups of mice (LP and HP) were chosen for the comparative transcriptome analysis. A total of 88 genes were found to be differentially expressed (*p*_adj_ < 0.1) between groups HP and LP (64 upregulated and 24 downregulated as compared to group LP; see the full list in Supplementary Table S1).

**Figure 3:**
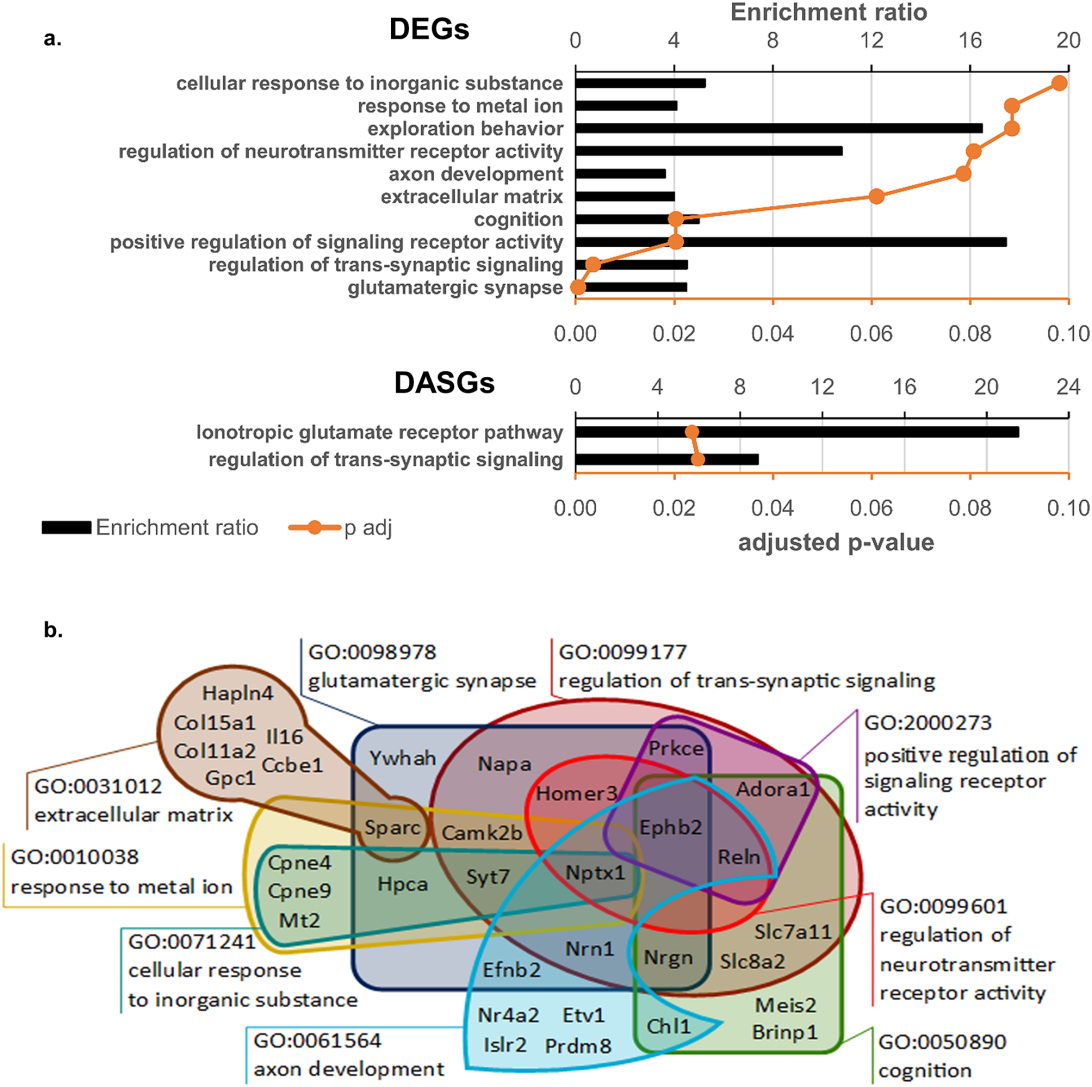
GO enrichment analysis of DEGs and DASGs. (**a**) GO terms overrepresented among DEGs and DASGs, FDR < 0.1. The top X-axis and histogram illustrate the enrichment ratio, whereas the bottom X-axis and orange curve denote significance of the changes. (**b**) A representative diagram illustrating overlaps of the sets of genes matching the enriched GO terms. GO term “exploration behavior” (GO:0035640, associated with genes *Brinp1*, *Chl1*, and *Prkce*) is not shown in the figure.

The functional enrichment analysis was conducted in the WebGestalt database^20^. Although the number of identified genes was modest, we found 57 significantly enriched (FDR < 0.1) terms among biological processes, 19 terms among cellular components, and four among molecular functions (Supplementary Table S2). After filtering by the noRedundant WebGestalt option, 10 significantly enriched terms remained, with eight terms from biological processes and two terms from cellular components (Fig. 3a, Supplementary Table S2). It is worth noting that 70% of the DEGs (62 of 88) matched one or another enriched GO term, whereas 38% of the DEGs (33 of 88) matched enriched noRedundant GO terms. This finding indicates strong functional relatedness of the identified DEGs. Detailed analysis of GO enrichment showed (Fig. 3b, Supplementary Table S2) that the most enriched terms were “positive regulation of signaling receptor activity” (R [ratio of enrichment] = 17.5, *p*_adj_ = 0.0203), “regulation of neurotransmitter receptor activity” (R = 10.8, *p*_adj_ = 0.0807), and “exploration behavior” (R = 16.5, *p*_adj_ = 0.0885), which are included in a more comprehensive term “regulation of trans-synaptic signaling” (R = 4.5, *p*_adj_ = 0.0035; Fig. 3b) and involve genes whose products participate in modulation of the frequency, rate, or extent of signal transduction. The last term is tightly connected with term “glutamatergic synapse” (R = 4.5, *p*_adj_ = 0.0005), and the overlap of these two gene sets includes nine genes (*Nrn1*, *Nptx1*, *Ephb2*, *Homer3*, *Prkce*, *Napa*, *Camk2b*, *Syt7*, and *Nrgn*) that take part in the regulation of synaptic signaling in glutamatergic neurons. The most interesting finding—directly related to the conducted tests—is enrichment of the term “cognition” (R = 5.0, *p*_adj_ = 0.0203). DEGs matching this term are broadly involved in trans-synaptic signaling, including genes participating in the transport of the calcium ion (*Slc8a2*) and glutamate (*Slc7a11*), adenosine (*Adora1)* and ephrin receptors (*Ephb2*), calmodulin-binding protein (*Nrgn*), and a serine protease regulating microtubule function in neurons (*Reln*). Besides, this set includes genes specifically associated with hippocampal memory formation (*Brinp1*, *Chl1*, and *Meis2*)^21–23^. The other enriched GO terms are also partially associated with trans-synaptic signaling and the glutamatergic synapse and include “response to metal ion” (R = 4.1, *p*_adj_ = 0.0884) and “cellular response to inorganic substance” (R = 5.2, *p*_adj_ = 0.0981) which are mostly involved in the response to the calcium ion, as well as “axon development” (R = 3.6, *p*_adj_ = 0.0786) and “extracellular matrix” (R = 4.0, *p*_adj_ = 0.0610).

### Differences in alternative splicing events are associated with the glutamate receptor pathway

In the analysis of alternative splicing using the rMATS tools, we excluded low-coverage events (fewer than 10 counts per inclusion and skipping event counts). By doing so, we detected 14 differential alternative splicing events between groups HP and LP. The splicing events included one alternative 3′ splice site, two alternative 5′ splice sites, eight skipped exons, and three mutually exclusive exons, thus totaling 12 affected genes (Supplementary Table S3). Parallel analysis by the DEXSeq tool revealed 12 other genes with alternative exon usage (Supplementary Table S4). These programs utilize different algorithms for assessing alternative splicing events and therefore may complement each other^24^. In subsequent analyses, we used a combined list of the 24 differentially alternatively spliced genes (DASGs).

Overall, among the 26 identified splicing events, nine events were related to changes in either the 5′ untranslated region or 3′ untranslated region, and the others affected the coding part of a gene. Changes in the coding part of a gene may give rise to various protein isoforms with different functional properties. Among the DASGs, there were three transcripts—*Gria1*, *Gria2*, and *Snap25*—whose protein isoforms are well characterized.

Alternative splicing generates two isoforms, “flip” and “flop,” of AMPA receptor subunits GRIA1 and GRIA2. These isoforms differ by a five amino acid residues located within a conserved receptor domain, which forms a part of the extracellular M3–M4 loop^25^. In the HP group, there was lower prevalence of the “flop” splice variant of the *Gria1* transcript (NM_008165.4, ENSMUST00000094179.10) and simultaneously higher prevalence of the “flip” splice variant of *Gria1* (NM_001113325.2, ENSMUST00000036315.15) as compared with the LP group. For gene *Gria2*, there was only higher abundance of the “flip” splice variant of the *Gria2* transcript (NM_001083806.3, ENSMUST00000107745.7) in the HP group than in the LP group. Furthermore, because the flip-flop isoforms are known for all four subunits of AMPA receptor, we tested the ratio of the splice variants for genes *Gria3* and *Gria4*. It turned out that in both cases, there was a tendency for a higher proportion of the “flip” splice variant in group HP (*p* = 0.029 and *p* = 0.013, respectively), but this difference did not persist after the adjustment for the multiple comparison. Nonetheless, we can say that there was an obvious trend: a greater proportion of the “flip” splice variant of all four genes encoding AMPA receptor subunits.

Among splice variants of *Snap25* (synaptosome-associated protein 25, which participates in synaptic vesicular transport), there were functional splice variants with mutually exclusive exons 5a and 5b, which code for the membrane-interacting domain^26^. Splice variant *Snap25b* (NM_001291056.1, ENSMUST00000110098.3) was less prevalent in the HP group than in the LP group.

It should be noted that all the genes whose transcripts were found to be affected by alternative splicing were not DEGs, i.e., a significant shift in splice variant expression did not change the expression levels of these genes.

Next, we conducted functional GO enrichment analysis of the DASGs and found significantly enriched terms from the category “biological processes” that are related to long-term memory (GO:0007616 [*Gria1*, *Shank1*, and *Snap25*], *p*_adj_ = 0.0248), regulation of trans-synaptic signaling (GO:0099177 [*Gria1*, *Nptn*, *Rhot1*, *Shank1*, *Snap25*, and *Ssh1*], *p*_adj_ = 0.0248), and the ionotropic glutamate receptor pathway (Panther pathway P00037 [*Gria1*, *Shank1*, and *Snap25*], *p*_adj_ = 0.0236) (full list in Supplementary Table S5).

Consequently, most genes whose expression differs between groups HP and LP are associated with trans-synaptic signaling and the signaling pathway of the glutamatergic synapse. We chose genes participating in these processes for validation of the RNA-seq results by PCR.

### qPCR validation

To validate RNA-seq data on a larger number of samples (n = 6 for the LP group and n = 10 for the HP group), we performed qPCR analysis on genes *Nptx1*, *Slc8a2*, *Syt7*, *Nrgn*, *Napa*, and *Camk2b*. Proteins coded by these genes are related to the glutamatergic synapse and AMPA receptor pathway.

We confirmed that the expression of neuronal pentraxin *Nptx1* and Na/Ca transporter *Slc8a2* is greater in the HP group (*p* < 0.01, Fig. 2b). Meanwhile, the expression levels of the other genes were not different between the groups. Addition of samples insignificantly increased variation in a group (see barplots with error bars in Fig. 2b); therefore, confirmation of expression differences by PCR for only 2 out the 6 selected genes can be explained by only a difference in sensitivity of the methods being used. By PCR, we were able to confirm the change in the expression of target genes that featured the largest expression fold changes (FC = 1.29 for Nptx1 and FC = 1.25 for Slc8a2); the other genes showed small fold changes of expression (from 1.14 to 1.22). Consequently, we were unable to confirm the significant differences in expression because of the limitations of the PCR method.

Despite fairly low values of the difference in the inclusion level (IncLevelDifference, Supplementary Table S3), we noticed that the “flop” splice variant of the *Gria1* transcript tended to be underexpressed in the HP group (*p* = 0.077), resulting in a significantly higher flip/flop ratio of the splice variants in the HP group than in the LP group (*p* = 0.032, Fig. 2c). For splice variant *Snap25b*, we also detected only marginally significant downregulation of this transcript in the HP group compared to the LP group (*p* = 0.077). Analysis of the other transcripts did not reveal significant differences between the groups.

### WGCNA expands the network of genes associated with learning performance

**Figure 4:**
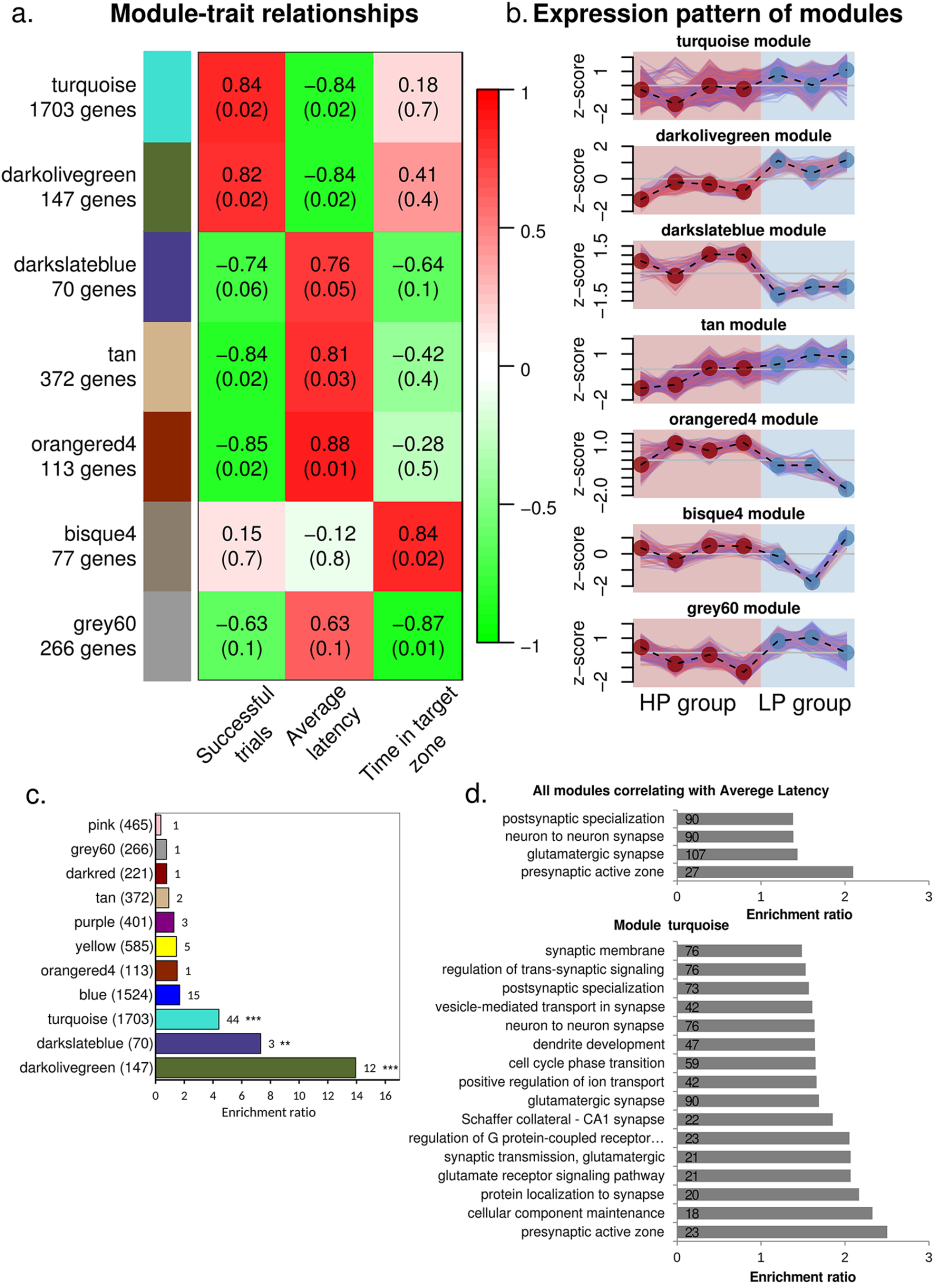
Functional characterization of co-expression modules. (**a**) The heatmap of module–trait relationships for behavioral data. Only modules significantly correlating with behavioral traits are shown. Cell color and number represent the coefficient and *p* value of Pearson’s correlation. Module names are accompanied by the number of corresponding genes. (**b**) The expression pattern of modules. The y-axes represent the z-score of rLog-transformed counts. Lines indicate a scaled expression pattern of each gene. The dashed line indicates the mean value of the expression. Point colors correspond to experimental groups. (**c**) The bar plot illustrating the enrichment of gene modules with DEGs. Numbers in bars indicate the found DEGs. Values > 1 mean enrichment of this module with DEGs, and values < 1 denote depletion of DEGs. Fisher’s test, ***p < 0.001, **p < 0.01. (**d**) GO term enrichment analysis of all modules that showed high correlation with the average latency to find the platform, and below, the data from separate GO analyses of the turquoise module. The number of genes in a category is shown inside each plot. Both analyses identified enrichment of the terms related to the glutamatergic synapse.

Because DEG analysis detects only substantial differences in expression, we analyzed the gene co-expression network to reveal the genes tightly connected to the already detected DEGs. The WGCNA yielded 56 co-expressed modules each containing 32–1703 genes (Supplementary Table S6). Among them, expression changes in five modules (covering 2405 genes) correlated with changes in the behavioral parameters “the average latency to find the platform” and “the total number of successful attempts,” whereas gene expression changes in two modules (343 genes total) correlated with changes in “time spent in the target sector” (Fig. 4a,b). DEGs were present in various modules, with the largest numbers in modules “turquoise,” “blue,” and “darkolivegreen” (Fig. 4c).

We then determined which processes are related to the genes present in the modules correlating with behavior. According to the GO enrichment analysis, the set of genes whose expression correlated with “the average latency to find the platform” is enriched mostly with genes related to synapse function (149 genes, 6.2%) (GO terms: “glutamatergic synapse,” “neuron to neuron synapse,” “postsynaptic specialization,” and “presynaptic active zone”; Fig. 4d). More detailed examination revealed that these genes were mostly concentrated in the largest module (turquoise), which also contained a half of our DEGs.

GO terms that were found to be enriched in the turquoise module are related to synaptic signaling, vesicular transport, and ionic permeability of a synaptic membrane. It was this module that contained many genes (90) whose products are associated with glutamatergic synapse function (GO:0098978, FDR = 0.0025), and some of them were significantly differentially expressed between the studied groups.

### Cell type enrichment

We performed the cell type enrichment analysis using cell type–specific marker lists previously identified in five purified brain cell types: neurons, astrocytes, microglia, oligodendrocyte precursor cells, mature oligodendrocytes, and endothelial cells^27^.

**Figure 5:**
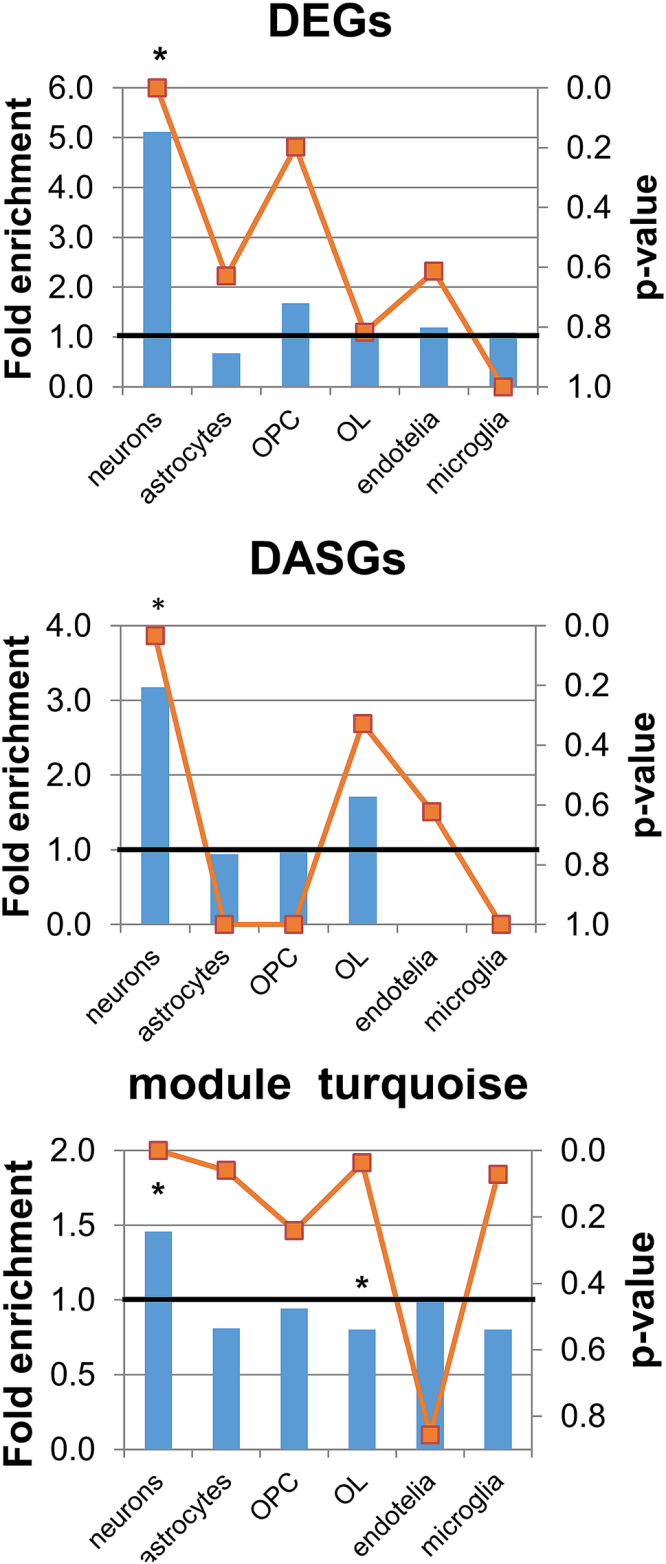
Cell type enrichment analysis. The left Y-axis and histogram illustrate fold enrichment by cell type, whereas the right Y-axis and the orange line denote significance of the changes. *p < 0.05 as compared with a chance occurrence. *OPC* oligodendrocyte precursor cells, *OL* mature oligodendrocytes. The sets of DEGs and DASGs and the turquoise module are more enriched with neuron-specific genes than expected by chance.

The analysis of cell type enrichment across the network revealed that the turquoise module, which correlated negatively with “the average latency to find the platform,” was enriched with neuron-specific genes (E = 1.46,* p* = 1.67 × 10^−6^, Fisher’s exact test) and was depleted of others, especially oligodendrocyte-specific genes (*p* = 0.038, Fisher’s exact test; Fig. 5). Our DEGs and DASGs were also found to be enriched with neuron-specific genes (E = 5.11,* p* = 3.07 × 10^−13^, and E = 3.17, *p* = 0.034, respectively; Fisher’s exact test).

### Identification of a master regulator

Analysis of consensus target genes for TFs with data from multiple experiments (ENCODE and ChEA Consensus TFs from ChIP-X, EnrichR) identified two potential TFs (REST and SUZ12) for the DEGs and neuron-specific DEGs, and three TFs (REST, UBTF, and ZBTB7A) for genes of the turquoise cluster (Supplementary Table S7). REST had the highest combined score both according to ChEA and ENCODE data and showed enrichment with DEGs (OR = 4.13 for ChEA data and OR = 5.4 for ENCODE data), with neuron-specific DEGs (OR = 8.41 for ChEA data and OR = 10.4 for ENCODE data), and with genes of the turquoise cluster (OR = 1.53 for ChEA data and OR = 1.9 for ENCODE data). Meanwhile, enrichment analysis of the input gene set (15,028 genes) did not detect specific enrichment for this REST TF (OR = 1.05 and OR = 1.07, respectively). Analysis via HOCOMOCO models yielded 128 potential REST-binding sites in 62 DEGs (70%), and 2417 such sites in 1230 genes of the turquoise cluster (72%) (Supplementary Tables S8 and S9). A comparison of the REST targets identified on the basis of ChIP-seq data (ChEA and ENCODE) and the genes containing a REST-binding motif (HOCOMOCO v11) yielded 20 DEGs that may be considered most likely targets of REST (Supplementary Table S10). GO enrichment analysis of these 20 genes revealed four significantly enriched terms from the “biological processes” category (GO:0035640 “exploration behavior” [*Brinp*, *Chl1*, and *Prcke*], padj = 0.0006; GO:0010038 “response to metal ion” [*Camk2b*, *Cpne4*, *Cpne9*, *Hpca*, and *Syt7*], padj = 0.0183). There were enriched terms from the “molecular processes” category GO:0003779 “actin binding” [*Enc1*, *Hpca*, *Ncald*, *Prkce*, and *Ywhah*], padj = 0.0391).

## Discussion

The MWM test is a well-established test of spatial learning and long-term memory^28,29^. Long-term memory is defined as storage of information for a specific experience or event for prolonged time. Acquisition and consolidation of long-term memories is associated with such processes as long-term potentiation (LTP), long-term depression (LTD), and spike timing dependent plasticity^30–32^, which require de novo mRNA and protein synthesis^33,34^. Performance on the MWM task has been shown to be highly dependent on hippocampal function because hippocampal lesions impair acquisition during training trials and subsequent probe trial performance^35,36^. At the molecular level, training in the MWM test activates hippocampal molecular changes including a redistribution of glutamate receptors, activation of protein kinases, and alterations in gene expression^37–41^.

In this study, we focused on changes in the expression levels of genes and compared hippocampal transcriptome data between mice with high and low cognitive performance in the MWM. We identified 88 DEGs between HP and LP mice and noticed a significant difference in isoform usage for 24 more genes. Further analysis revealed that the identified genes are functionally connected with one another and match GO terms related to the regulation of trans-synaptic signaling, cognition, and glutamatergic transmission. These findings were supported by the results of WGCNA, which showed that modules of co-expressed genes that correlate with MWM cognitive performance were also enriched with similar GO terms. Accordingly, as a consequence of learning and formation of long-term memory in the MWM test, the events ensuring changes in the expression of genes from the same functional groups may take place at different levels of gene expression regulation.

Changes in the expression of genes in relation to the learning in the MWM test have been previously investigated in several studies. For instance, in a comparison of well and poorly MWM-performing strains of mice by microarray analysis, researchers found 27 DEGs in the hippocampus^41^. A study on a cognitive deficit in the MWM test involving aged rats revealed ~ 300 DEGs (as evidenced by microarray analysis) between cognitively intact and cognitively impaired aged rats in various hippocampal zones^42^. Exposure to chronic variable stress causes changes in MWM performance in mice, and transcriptome analysis by RNA-seq revealed multiple genes (more than 1200) and pathways that are significantly associated with this cognitive modification^43^. Because of differences in experimental design and in methods of expression analysis, the identified sets of genes associated with good spatial learning rarely overlap among such studies. The largest discrepancies in results are caused by the period after which hippocampal samples are collected. For example, in a study on cognitive abilities of aged rats, the levels of gene expression were evaluated 1 week after the MWM test^42^, whereas in a study on the effects of chronic stress, this evaluation was performed 10 min after the probe trial in the MWM test^43^. In our experiment, we chose the 1-day interval after the probe trial in the MWM. It is reported that for stabilization of a memory trace and for formation of long-term memory, a certain period (from several hours to several days) is necessary because these processes involve protein synthesis and synaptic changes^44^. The processes of synaptic consolidation are accompanied by post-translational modifications, modulation of gene expression, and the synthesis of gene products that alter synaptic efficacy^45^. For this reason, the proposed (by us) 24 h period after the probe trial matches the period necessary for the processes of consolidation of long-term memory. Furthermore, this approach minimizes the effects related to the acute stress of exposure to the MWM^46^. Accordingly, our experimental design is probably more suitable for the identification of genes associated with long-term memory, whereas at 10 min after the end of the testing, either mostly short-term memory or a combination of short-term memory and long-term memory is evaluated.

The largest number of interconnected changes in the transcriptome that we detected by various analytical methods (differential gene expression analysis, analysis of alternative splicing events, and WGCNA) is related to changes in the homeostasis of glutamatergic synapses. First of all, we should highlight the significant difference in isoform usage for genes *Gria1* and *Gria2* (and a marginally significant difference for genes *Gria3* and *Gria4*) among alternative splicing events: higher production of the “flip” splice variant and lower production of the “flop” splice variant in the HP group than in the LP group. The “flip” isoform of the protein is desensitized more slowly and recovers from desensitization more rapidly, but AMPA receptor affinity for glutamate remains unchanged^47–49^. Besides, it is known that the ratio of these isoforms depends on the activity of cells: chronic activity deprivation by a Na^+^-channel blocker shifts the ratio of the alternatively spliced transcripts toward the “flop” splice variant, at least in the CA1 subfield of the hippocampus^50^. It can be hypothesized that the expression of AMPA receptors showing altered kinetics will enhance the traffic of Na^+^ ions through the AMPA receptors in the glutamatergic synapse and should result in higher postsynaptic efficacy in the HP group, thereby contributing to more effective formation of long-term memory. It is noteworthy that the expression levels of genes of the AMPA receptors themselves were not different between the groups of mice, and these genes were not found in any gene modules correlating with learning, according to our WGCNA data.

The activity of the AMPA receptor complex may be additionally modulated by accessory subunits such as transmembrane AMPA receptor regulatory proteins (TARPs) and cornichon family (CNIH) proteins^51,52^, which also take part in the trafficking of AMPA receptors to postsynaptic densities. In our WGCNA, most genes of TARPs (γ-2, 3, 6, 7, and 8) and *Cnih2* showed coordinated expression and ended up in the turquoise module, which correlates with the learning ability, and these genes were slightly upregulated in the HP group (judging by the nominal *p* value). Aside from TARPs, many other proteins participate in the scaffolding of AMPA and NMDA receptors and in synaptic-vesicle trafficking of AMPA receptors and are all members of the pool of postsynaptic density (PSD) proteins. We extracted a list of PSD proteins that were detected in mouse synapses in Ref.^53^ and found that 25% of our DEGs encode proteins that are components of the PSD of neurons (OD = 3.2,* p* = 1.772e−05, Fisher’s test), and the expression of most of them is higher in the well-trained mice (HP group). Additionally, the prevalence of such genes is high in WGCNA modules correlating with learning (OD = 1.33, *p* = 7.733e−05, Fisher’s test).

We noted higher expression of the *Camk2b* gene coding for protein kinase CaMK2β, which directly phosphorylates AMPA receptor subunits, thus increasing channel conductance and promoting LTP^54^. Our study also shows that in the HP group, the expression of *Nptx1* (neuronal pentraxin 1, Np1) and *Nrgn* (postsynaptic protein neurogranin, Ng) was higher, which are important components of AMPA receptor clustering machinery. Np1 selectively accumulates at excitatory synapses and mediates synaptic recruitment of AMPA receptors^55,56^. Ng regulates calmodulin distribution within dendritic spines, and its overexpression facilitates LTP^57^. Besides, low Ng levels correlate with poor performance on the MWM test^58^.

We can theorize that the greater necessity of the mRNA of these and other genes of PSD indicates greater consumption of proteins in active glutamatergic synapses and expenditures for the maintenance of LTP/LTD, which are necessary for learning and for the formation of long-term memory. Differences were found in vesicular traffic too: there was upregulation of genes participating in the release of mediators from the presynaptic membrane and of genes taking part in the traffic of AMPA receptors from a readily releasable pool of synaptic vesicles. We observed upregulation of genes *Napa* (NSF-attachment protein alpha) and *Syt7* (synaptotagmin 7), whose products are important components of the SNARE complex involved in the delivery of AMPA receptors to the synapse^59,60^. The enhanced expression of *Napa* seen in the HP group is suggestive of greater velocity of NSF-mediated disassembly of the SNARE complex^61–63^. The velocity of SNARE complex disassembly in turn determines the velocity of neuronal exocytosis and efficiency of synaptic transmission^63^. For another component of the SNARE complex, which is encoded by *Snap25*, we documented a difference in the ratio of transcripts *Snap25a* and *Snap25b*, namely, in the HP group the prevalence of *Snap25b* was lower than that in group LP. It is reported that SNAP25b supports a larger primed-vesicle pool than SNAP25a does, thereby giving rise to a two- to threefold difference in the size of the exocytotic burst^64^. Overall, the mice devoid of isoform SNAP25b have a much worse learning ability, but some tasks during learning in an active avoidance spatial learning task are performed faster by SNAP25b-deficient mice than by mice with the normal ratio of the isoforms^65^. Of note, the SNARE complex participates not only in postsynaptic AMPA receptor trafficking but also in a neurotransmitter release at presynaptic membranes of glutamatergic and GABAergic neurons. This observation points to a complicated influence of the Snap25a/b isoform ratio on the level of synaptic activity^66^, and we cannot unambiguously interpret the downregulation of *Snap25b* in a context of long-term memory formation. Nevertheless, our data suggest that changes in the expression of genes encoding proteins of the SNARE complex (*Syt7*, *Napa*, and *Snap25*) may be an adaptation to a faster neurotransmitter release from synaptic vesicles^67^. In addition, a substantial number of our DEGs code for proteins playing a part in the turnover of calcium (*Hpca*, *Caln1*, *Orai2*, *Cpne4*, and *Cpne9*), which acts as a secondary messenger triggering complex signaling cascades.

**Figure 6:**
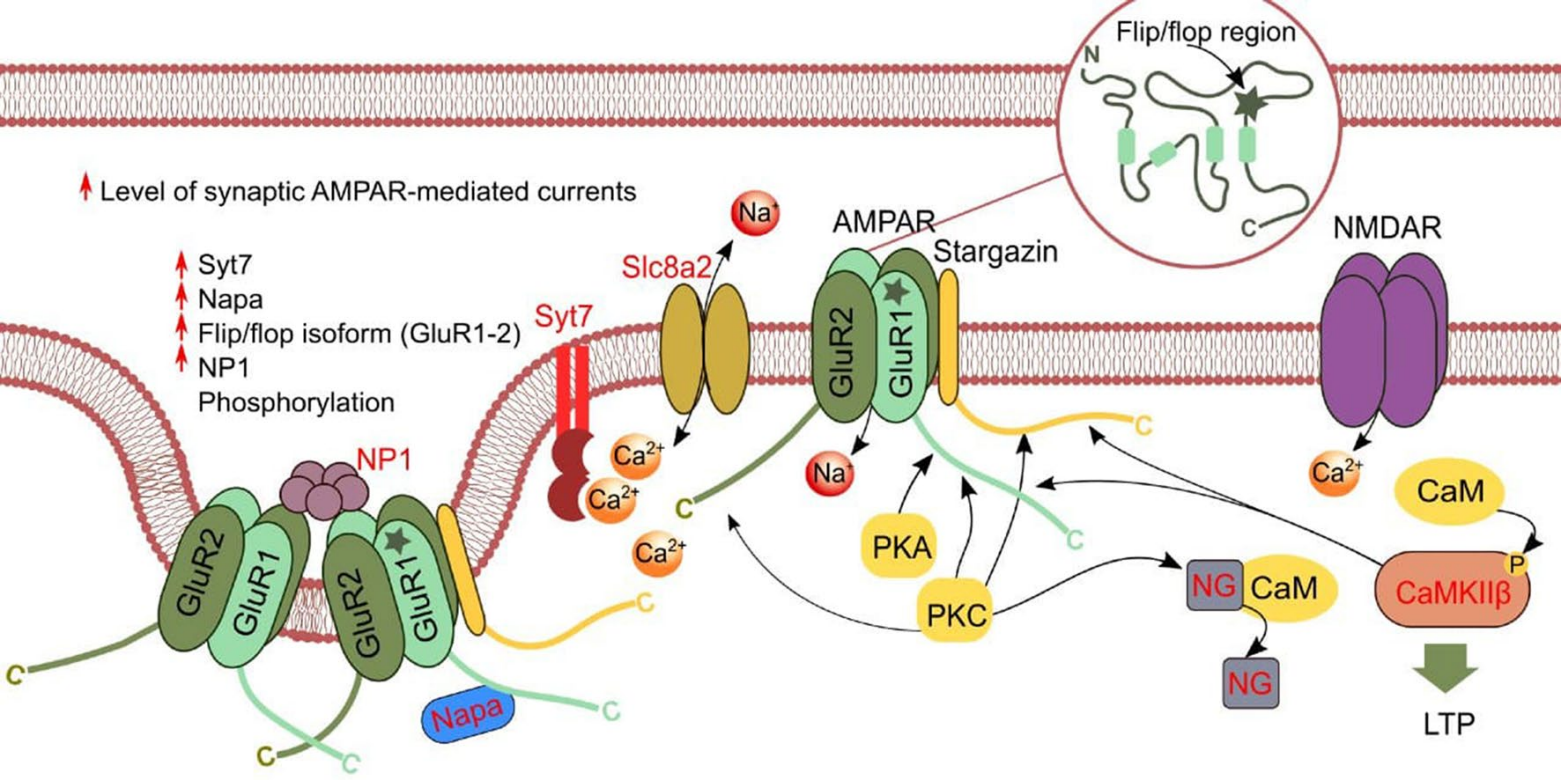
Schematic diagram illustrating the role of proteins encoded DEGs and DASGs and others on AMPA-receptor signaling and synaptic plasticity. mRNA expression of NSF-attachment protein alpha (*Napa*), pentraxin 1 (*Nptx1*, NP1), synaptotagmin 7 (*Syt7*), neurogranin (*Nrgn*, Ng), *Slc8a2*, and *Camk2b* was higher in the HP group (corresponding to the proteins highlighted in red). The HP group also has an increased flip/flop transcript ratio of subunits Glur1 and Glur2. The flip isoform renders AMPA receptors less prone to desensitization as compared to the flop isoform, but AMPA receptor affinity for glutamate remains unchanged^48,91^. Fast, excitatory synaptic transmission in the brain is primarily mediated by two types of ionotropic glutamatergic receptors: AMPA and NMDA receptors. Calcium entry through postsynaptic NMDA receptors activates intracellular signaling cascades including calmodulin (CaM) and CaMK2 signaling. Ng concentrates CaM to participate in postsynaptic signaling processes^92,93^. Ng phosphorylation by PKC disrupts its binding to CaM, thereby leading to the activation of the CaMK2β signaling pathway and induction of LTP. CaMK2β potentiates ion channel function of AMPA receptor via direct phosphorylation of AMPA receptor subunits^94^. Another kinase, PKA, phosphorylates the C-terminal domain of AMPA receptor subunits and thus controls synaptic trafficking that underlies plasticity^95^. Syt7 is essential specifically for Ca^2+^-induced AMPA receptor recruitment during LTP^60^ and contributes to the rapid decrease of cytoplasmic Ca^2+^ levels back to baseline after neuronal activation, thereby contributing to the modulation of synaptic plasticity^96^. NP1 drives the clustering of AMPA receptors at the postsynaptic membrane, promotes excitatory synaptogenesis, and mediates synaptic recruitment of AMPA receptor^55,56^. Thus, phosphorylation of AMPA receptor mediated by CaMK2β as well as an increased level of Napa, Nptx1, and Syt7 and the increased flip/flop isoform ratio of GluR1 and GluR2 during LTP potentiate ion channel function, cause desensitization, and increase the levels of synaptic AMPA receptors.

Taken together, our findings point to an important role of increased expression of the identified genes in AMPA receptor scaffolding and in the regulation of AMPA receptor trafficking to a synapse, thereby possibly ensuring better MWM cognitive performance (Fig. 6). The importance of glutamatergic transmission for manifestation of cognitive abilities is highlighted by a finding that most mutations (in subunits of the relevant receptor or in its accessory subunits) that worsen the functioning of the glutamatergic system are linked with intellectual disability^68^.

It is known that spatial learning tasks facilitate the implementation of persistent hippocampal LTP or LTD, suggesting a tight association between synaptic plasticity and hippocampus-dependent learning^69^. AMPA receptors mediate most of the fast synaptic transmission in the mammalian brain. Trafficking of AMPA receptors (rapid changes in the number of receptors in PSD) and the regulation of ion channel function play a key role in learning-facilitated synaptic plasticity^70^. On the basis of our data, we can hypothesize that high MWM cognitive performance in mice is linked with the facilitation of synaptic plasticity (LTP/LTD induction) that is mainly mediated by enhanced glutamatergic transmission, primarily AMPA-receptor signaling.

Finally, we found that a TF called REST is a possible master regulator of the identified DEGs and is probably related to hippocampus-dependent learning. Promoter regions of > 70% of our DEGs and neuron-specific DEGs contain binding sites for this TF. REST is a key modulator of the neuronal epigenome, and REST’s downstream genes are involved in neuronal differentiation, axonal growth, vesicular transport, ion channel conductance, and synaptic plasticity^71^. Recent evidence suggests that REST not only silences but also activates target genes^72^. REST takes part in transcription regulation of a number of genes involved in learning and memory processes^73,74^, in particular, REST exerts control over the transcription of *Arc, Egr1*, and *Bdnf*^75,76^. Nonetheless, we did not notice differences in *Rest* expression between groups HP and LP. This result does not rule out the role of REST as a master regulator of genes associated with spatial learning. This is because implementation of such effects may involve not only the levels of REST expression but also post-translational modifications of REST as well as various cofactors necessary for this protein’s function^71^.

A massive search for the genes associated with learning and memory has been conducted in the last decades. The data obtained by the microarray technology have helped to identify potential clusters of genes tentatively related to the processes of learning and memory in mice^41^ and in rats^77,78^. The development of more precise methods for studying the transcriptome, e.g., RNA-seq, has propelled this research field to a new level of searching for the genes that encode proteins involved in the processes of memory consolidation, in no small part due to the additional ability to analyze alternative splicing events.

Our results indicate the importance of glutamatergic transmission for effective learning during tasks that require spatial memory. Most of all, these phenomena are relevant to the activity of the AMPA receptor signaling pathway, which launches a cascade of reactions contributing to persistent LTP. We cannot say with certainty whether these changes are linked with the formation of long-term spatial memory during learning in the MWM test or whether the high performance on the MWM test is caused by preexisting shifts in glutamatergic neurotransmission in HP mice. We saw a difference in the gene expression profile at a certain time point and suppose that the higher level of transcription is accompanied by greater protein synthesis. Numerous studies indicate that it is new protein synthesis that is needed for long-term storage of memory and for the maintenance of long-term plasticity^33,34^. In the future, to determine whether the observed changes are a universal phenomenon, it is necessary to conduct similar experiments on other mouse cohorts, of different sex or strain.

## Methods

### Ethics statement

All experiments were conducted according to the European Union Directive 2010/63/EU for animal experiments and further approved by the Ethical Committee of the Institute of Cytology and Genetics, SB RAS (Protocol #25, December 2014). In this study, all methods were performed in accordance with the relevant guidelines and regulations.

### Animals

C57BL/6 mice were housed at the Center for Genetic Resources of Laboratory Animals, the Institute of Cytology and Genetics (SB RAS, Novosibirsk, Russia, RFMEFI62119X0023). The animals were housed under standard conditions (a 12/12 h light/dark cycle, lights on at 8:00 a.m.; feed [pellets] and water available ad libitum).

### The MWM test

This test was carried out to assess hippocampus-dependent spatial long-term memory and learning^35^. Forty-seven adult male mice at 12–14 weeks of age were used in the experiment. The animals were individually placed into a new cage 2 days before the test. The water maze consisted of a circular tank (diameter = 1 m; height = 30 cm) that was filled with tepid water (23 ± 1 °C) that was made opaque by the addition of powdered milk. The circular tank was divided into four equal sectors (Target, Opposite, Sector 1, and Sector 2), each with a spatial cue on the tank wall. A white escape platform (diameter = 10 cm, height = 10 cm) was located 1 cm below the water. The water temperature was 24 ± 1 °C. The testing lasted for 5 days. First, mice were trained for 4 consecutive days and underwent four training trials per day, for a total of 16 training trials, each starting at the same time of day. In each training trial, a mouse was placed in one of the sectors and allowed to search for the hidden platform for 1 min. If the mouse did not find the platform within 1 min, the experimenter led the animal to it. After the platform was located, the mouse was left on it for 15 s to memorize the spatial cues. After that, the mouse was placed in a cage for 15 s for resting before the next trial. Throughout the experiment, the platform remained at its original position. To assess the learning ability, latency to find the platform in each trial and the total number of successful attempts were registered. Finally, on day 5, a probe trial was administered: the platform was removed, the mouse was placed in the Opposite sector, and the time spent in each sector within 1 min was measured. The test data were recorded and processed using the EthoStudio software^79^. On the basis of parameters “average latency to find the platform” and “the number of successful attempts”, the mice were subdivided into two groups: high cognitive performance in the MWM (HP group) and low cognitive performance in the MWM (LP group). Details of the group assignment are given in the “Results” section.

### Tissue collection and RNA extraction

Mice were killed by decapitation between 10:00 AM and 12:00 PM the next day after the probe trial in the MWM test. Trunk blood was collected, and serum was prepared by centrifugation at 3000×*g* for 10 min at room temperature and stored at − 80 °C until use. The whole hippocampus was quickly excised and frozen in liquid nitrogen and then stored at − 80 °C until analysis. RNA was extracted from frozen tissue with the TRI-Reagent (Sigma, USA) according to the manufacturer’s instructions. The total RNA was purified on Agencourt RNAClean XP beads (Beckman Coulter, Germany). The quality and quantity of total RNA were evaluated on a NanoDrop 2000 spectrophotometer. The quality of samples for RNA sequencing was assessed by means of an Agilent 2100 Bioanalyzer and the Total RNA Nano Kit (Agilent Technologies, USA). Only samples with an RNA integrity number greater than 8.0 were used for gene expression analysis.

### Library construction

For sequencing, we selected animals from groups differing in behavior (LP and HP) according to the following criteria: mice should come from different litters (not siblings) and behavioral parameters in the MWM should reflect the direction of changes in the whole group. Four mice from each group were chosen for RNA-seq. RNA-seq libraries of the murine hippocampus were prepared in accordance with the standard New England Biolabs protocol used earlier in our lab^80,81^. Briefly, polyA-tailed mRNAs were purified from 1 µg of total RNA using the NEBNext Poly(A) mRNA Magnetic Isolation Module. Then, directional cDNA libraries were created by means of the NEBNext Ultra II Directional RNA Library Prep Kit for Illumina. Size selection of DNA fragments was performed on Agencourt AMPure XP beads (Beckman Coulter, USA). Next, PCR enrichment of the adapter-ligated library was conducted (six cycles of PCR). The size and quantity of the library were verified on the Agilent Bioanalyzer, and libraries were subjected to paired-end (2 × 100) sequencing on the Illumina HiSeq 4000 platform (Evrogen Joint Stock Company, Russia). One library from the LP group did not pass the quality control after the sequencing and was excluded from further processing.

### Gene expression analysis

On average, ~ 30 million paired-end reads (21–36 million) were obtained from each sample by Illumina stranded sequencing. The sequencing data were preprocessed with the Trimmomatic 0.36 tool^82^ to remove adapters and low-quality sequences. The preprocessed data were mapped to the *Mus musculus* GRCm38 reference genome assembly in HISAT2 version 2.1.0^83^. The quality of the sequencing data was assessed using FastQC and Picard CollectRnaSeqMetrics tools (http://broadinstitute.github.io/picard/) (Supplementary Table S11). The libraries had average strand specificity above 97% and highly reproducible coverage bias. The aligned data with mapping quality (MAPQ) > 10 were then converted into per-gene and per-exon count tables by means of GENCODE vM13 gene annotation data. Genes with at least 10 counts in each sample were then subjected to an analysis of differential gene expression via the DESeq2 R-package^84^. Each per-exon count table was processed by means of the DEXSeq v1.30.0 package^85^ with default parameters to assess differential exon usage in pairwise comparison. In both cases, the Benjamini–Hochberg correction for multiple testing was applied to the resulting *p* values, and the genes with an adjusted *p* value (*p*_adj_) < 0.1 were designated as differentially expressed genes (DEGs).

### WGCNA

A gene co-expression network was constructed using the WGCNA package in the R environment^86^. Sequencing-depth–normalized gene level counts from the DESeq2 analysis served as input. Genes with low expression levels (< 10 counts in all samples) were filtered out, and rlog transformation was applied to the data on counts. A total of 15,028 well-expressed genes were chosen for generating the coexpression network. Scale-free topology fitting indices R^2^ were calculated for several soft threshold power values; when we selected a power value of approximately 0.8, it meant that the topology of the network was scale-free, and there were no batch effects. A signed weighted-gene correlation network was built by calculating a coefficient of correlation within all gene pairs. Then, using the soft threshold, the adjacency matrix was created. The corresponding dissimilarity was calculated from a topological overlap matrix that was obtained by transformation of the adjacency matrix. To identify gene modules, we employed average linkage hierarchical clustering and dynamic tree cut methods. The minimum module size was 30 genes. Highly similar modules were identified by clustering and then merged together with a height cut-off of 0.25. A total of 56 different modules were generated and assigned to colors (Supplementary Table S6). The correlation between modules and behavioral parameters was evaluated by Pearson’s correlation tests, and modules with *p* < 0.05 were considered significantly correlating.

### Cell type composition analysis

To address technical limitations such as the inability to physically break down the tissue into constituent cell types and analyze them separately, we sought to estimate whether any specific cell type contributed to the DEG list more than others did. For this purpose, we used lists of cell type–specific genes from a recently published article^27^, the expression of these genes is high only in one cell type. Genes specific to neurons, astrocytes, microglia, endothelial cells, oligodendrocytes, and oligodendrocyte precursor cells were tested for enrichment in our dataset.

### Enrichment analysis of gene ontology (GO) terms

GO enrichment analysis was conducted using WEB-based GEne SeT AnaLysis Toolkit (WebGestalt)^20^. As a reference set, we utilized a gene list from our RNA-seq dataset with counts > 10 (15,028 genes). GO terms with a false discovery rate (FDR) < 0.1 were considered significantly enriched.

### Differential splicing analysis

We employed two approaches to the analysis of alternative splicing: event-based (rMATS) and exon-based (DEXSeq). Firstly, we applied rMATS (version 4.0.2)^87^ to identify differential alternative splicing events including a skipped exon (SE), retained intron (RI), alternative 5′ splice site (A5SS), alternative 3′ splice site (A3SS), and mutually exclusive exons (MXE). We excluded from the analysis the events with mean counts of “inclusion” or “skipping” events < 10. The statistical model of rMATS calculates a *p* value and FDR and measures the difference in the splice variant ratio of a transcript between two conditions. Secondly, we applied DEXSeq v1.30.0^85^, which is an “exon-centric” analysis that explicitly tests for differential exon usage. DEXSeq is based on the method of testing for the deviation of read counts on individual exons from the counts of the whole gene. Thus, the combination of the two approaches allows for a more complete analysis of differential alternative splicing.

### Quantitative PCR (qPCR)

To validate the RNA-seq results, the expression of selected up- or downregulated genes and DASGs was confirmed by qPCR. One microgram of the total RNA was subjected to the synthesis of cDNA using a reverse-transcription kit (Syntol, Russia) with a random hexanucleotide mixture as primers. All procedures were carried out according to the manufacturer’s instructions. Primer sets for each gene were designed in Primer-BLAST (NCBI) (Supplementary Table S12). The qPCR cycling conditions were 95 °C for 5 min followed by 38 cycles of 95 °C for 10 s and 60 or 63 °C for 30 s. All qPCRs were carried out in duplicate. A melting-curve analysis was performed at the end of each qPCR run. All qPCRs were conducted on the Bio-Rad CFX platform (Bio-Rad, Hercules, CA) by means of a qPCR kit with Eva Green I (Syntol, Russia). The qPCR data were analyzed by the ΔΔCt method and normalized to β-actin (*Actb*) as a reference gene.

### The search for master regulators

This search was performed by the Web-based annotation tool EnrichR (ENCODE and ChEA consensus transcription factors [TFs] from ChIP-X)^88^. TFs were predicted for three gene sets: DEGs, neuron-specific DEGs, and the WGCNA-derived “turquoise” cluster correlating with behavioral parameters, with “*p* value < 0 0.05” as the cut-off. To identify the exact location of binding sites for the predicted TFs, we detected the motif occurrences in the promoter regions (± 500 bp from a transcription start site) of genes from the gene sets using HOCOMOCO collection v.11^89^. As fixed-motif *p* values, we used the recommended thresholds of 0.0005^89,90^.

### The serum level of corticosterone

These levels were measured by the Corticosterone ELISA Assay (Enzo, New York, NY, USA). Briefly, 90 μl of 0.9% saline was added to 10 μl of each serum sample. The assay was performed according to the manufacturer’s instructions. Each sample was assayed in duplicate.

### Statistical analysis of behavioral data

The normality of distribution and homogeneity of variances of behavioral and physiology data were tested by the Shapiro–Wilk test and Levene’s test, respectively. For normally distributed data (physiological parameters and gene expression), Student’s *t* test was performed. The behavioral data were not normally distributed, and therefore nonparametric tests were chosen. The behavioral data were analyzed by the Kruskal–Wallis test in case of three groups, and pairwise comparisons were made using the Mann–Whitney *U* test. The Wilcoxon matched pairs test was carried out for a comparison of time between Target and Opposite sectors. For correlation analysis between behavioral data and gene expression, Spearman’s rank correlation analysis was conducted. The statistical calculations were done in the STATISTICA 8 software. Hierarchical cluster analysis was performed to categorize the samples into subgroups of similar patterns of behavioral data. The Euclidean distance and average linkage clustering were employed to construct a hierarchical cluster analysis dendrogram.

## Supplementary Information

Supplementary Information

## Data Availability

The sequence data were deposited in NCBI BioProject under accession number PRJNA641667.
